# Does the *AMPD1* C34T Polymorphism Influence Physical Performance in Elite Athletes?

**DOI:** 10.3390/genes17080935

**Published:** 2026-08-11

**Authors:** Daniel R. Martin, Georgina K. Stebbings, Shane M. Heffernan, Robert M. Erskine, Mark Antrobus, Jon Brazier, Sarah Lockey, Alex L. Jackson, Stephen Day, Liam Kilduff, Mark Bennett, Stuart M. Raleigh, Tom Cullen, Malcolm Collins, Yannis Pitsiladis, Peter Callus, Adam J. Herbert, Alun G. Williams

**Affiliations:** 1 School of Sport and Exercise Sciences, Institute of Sport, Manchester Metropolitan University, Manchester M1 7EL, UK; 19078752@stu.mmu.ac.uk (D.R.M.); g.stebbings@mmu.ac.uk (G.K.S.); jacksonalex204@gmail.com (A.L.J.); petercallusmalta@gmail.com (P.C.); 2 Applied Sports Science Technology and Medicine Research Centre (A-STEM), Faculty of Science and Engineering, Swansea University, Swansea SA1 8EN, UK; s.m.heffernan@swansea.ac.uk (S.M.H.); l.kilduff@swansea.ac.uk (L.K.); markbennettanalytics@gmail.com (M.B.); 3 School of Sport & Exercise Sciences, Liverpool John Moores University, Liverpool L3 3AF, UK; r.m.erskine@ljmu.ac.uk; 4 Institute of Sport, Exercise and Health, University College London, London WC1E 6BT, UK; 5 Faculty of Wellbeing, Education and Language Studies, The Open University, Walton Hall, Milton Keynes MK7 6AA, UK; mark.antrobus@open.ac.uk; 6 Department of Psychology, Sport and Geography, University of Hertfordshire, Hatfield AL10 9AB, UK; j.brazier2@herts.ac.uk; 7 Faculty of Health, Education, Medicine and Social Care, Anglia Ruskin University, Chelmsford CM1 1SQ, UK; sarah.lockey@aru.ac.uk; 8 NHS Education for Scotland, Westport 102, West Port, Edinburgh EH3 9DN, UK; stephen.day@nhs.scot; 9 School of Sport, Exercise and Rehabilitation Science, University of Hull, Hull HU6 7RX, UK; stu.raleigh@gmail.com (S.M.R.); t.cullen@hull.ac.uk (T.C.); 10 Health through Physical Activity, Lifestyle and Sport Research Centre (HPALS), Department of Human Biology, University of Cape Town, Cape Town 7700, South Africa; malcolm.collins@uct.ac.za; 11 Department of Sport, Physical Education and Health, Hong Kong Baptist University, Hong Kong SAR 999077, China; ypitsiladis@hkbu.edu.hk; 12 Department of Movement, Human and Health Sciences, University of Rome ‘Foro Italico’, 00135 Rome, Italy; 13 Research Centre for Life and Sport Sciences, Birmingham City University, Birmingham B5 5JU, UK; adam.herbert@bcu.ac.uk

**Keywords:** *AMPD1*, athletes, distance runners, rugby union

## Abstract

**Background/Objectives**: Adenosine monophosphate deaminase deficiency has been implicated in impaired exercise capacity. We examined whether the *AMPD1* C34T (rs17602729) genotype was associated with athlete status and performance in distance runners (DRs) and rugby union (RU) athletes. **Methods**: Participants included 621 elite male RU athletes, 666 elite/sub-elite male and female DRs, and 1029 male and female non-athletes (NA), all of European ancestry. Genotyping was performed using real-time PCR with TaqMan reagents. Genotype and allele frequencies were compared using χ^2^ tests, while performance data were analysed using Kruskal–Wallis H, Mann–Whitney U, one-way ANOVA, and *t*-tests, with Benjamini–Hochberg correction applied for multiple comparisons. **Results**: Genotype and allele frequencies did not differ between athletes and NA or between athlete groups. In all DRs, run times did not differ between genotypes, although participants with the CT genotype in the elite and elite male subgroups were up to 5% faster than CC and TT homozygotes. In RU, participants with the CC genotype played 13% longer per appearance than those with the CT genotype, while forwards and front five participants with the CC genotype played 13% and 26% longer per appearance than T-allele carriers, respectively. Front five T-allele carriers made 75% more clean breaks than the front five with the CC genotype despite playing for a shorter time, although this finding should be interpreted cautiously. **Conclusions**: In summary, while the *AMPD1* C34T genotype was not associated with athlete status, the CC genotype was associated with greater RU playing time per appearance in forwards, and the CT genotype was associated with superior performance in elite DRs.

## 1. Introduction

Exceptional performance within a sporting discipline reflects the interaction of the athlete’s physiology, psychology, and training environment [1]. While aspects such as deliberate practice, nutrition, and the use of ergogenic aids can account for interindividual variance in physical performance [2,3,4], it has long been established that athletic status and physiological adaptation to training have a strong genetic basis [5]. The human genome may provide an individual with a natural predisposition towards enhanced endurance or sprint/power performance, although some variants may exert antagonistic effects across these phenotypes [6].

Endurance running places a large demand on the aerobic energy system, although anaerobic metabolism also contributes [7,8,9,10,11]. A combination of maximal rate of oxygen uptake (VO_2max_), lactate threshold, and running economy influences running ability [12,13,14,15,16]. As such, the heritability of VO_2max_ has been the focus of heritability research [17,18,19]. Estimates derived from the HERITAGE Family Study indicate that the heritability of VO_2max_ and other endurance-based phenotypes is ~50% [17,18,20]. 

In contrast, strength/power events are heavily dependent on anaerobic energy systems, specifically the degradation of the phosphagen phosphocreatine and the oxygen-independent breakdown of glycogen [9]. These systems provide energy at the point of contraction and for ~120 s thereafter [21]. The rapid provision of adenosine triphosphate (ATP) is essential for achieving the large power outputs required for success in strength/power events [9]. As with endurance traits, several strength, speed, and skeletal muscle phenotypes have a high degree of heritability, including hand grip strength [22,23], elbow flexor and knee extensor isokinetic strength [22,24], and jumping ability [23,25]. A meta-analysis conducted by Zempo et al. [26] estimated the heritability of skeletal muscle phenotypes to be ~50%.

Rugby union (RU) athletes are often exposed to a uniquely diverse set of physiological demands. RU comprises distinct playing positions, each with specific physiological, technical, and anthropometric demands [27,28,29,30], commonly grouped into forwards and backs. Unlike endurance athletes, there are various, and often competing, physical attributes that elite RU athletes must develop. Physical robustness is required to withstand frequent collisions over the course of an 80 min game [27,31]. However, a large VO_2max_ is needed to facilitate the performance of repeated high-intensity efforts with limited recovery [32,33]. Despite not impairing VO_2max_ in absolute terms, body mass shares an inverse relationship with energy cost of running [34,35,36], thereby limiting the endurance capabilities of RU athletes. 

Nevertheless, the energy contributions during ball-in-play (BiP) periods of RU are largely anaerobic in nature [32]. Skeletal muscle strength and power are prerequisites for collision (tackles, rucks, etc.) and line break success, as well as for sprinting, jumping, acceleration, deceleration, and change-of-direction ability [27,37]. Analyses of elite RU matches show that BiP accounts for only ~45% of the total match duration, with each BiP period averaging ~30 s [38], demonstrating the intermittent and largely anaerobic nature of RU match play. However, in-game GPS metrics such as distance covered, number of accelerations, and collision rates all decrease per unit time during extended BiP periods [39], highlighting the importance of a well-developed aerobic system for RU athletes in extended passages of play. 

Adenosine monophosphate (AMP) deaminase is a key regulator of skeletal muscle energy metabolism and a major component of the purine nucleotide cycle [40]. During physical activity, ATP is hydrolysed into adenosine diphosphate (ADP) [41], and two ADP molecules are converted into ATP and AMP by myokinase, leading to AMP accumulation [42]. AMP deaminase catalyses AMP deamination into inosine monophosphate and ammonia [40,43,44], shifting the myokinase reaction toward ATP production [42]. The skeletal muscle-specific isoform of AMP deaminase is encoded by the *AMPD1* gene [45], located on chromosome 1 [46] and expressed predominantly in type II muscle fibres [47]. The rs17602729 polymorphism brings about a C-to-T transition in nucleotide 34 in exon 2 of the *AMPD1* gene, converting a glutamine codon to a premature stop codon and reducing skeletal muscle AMP deaminase activity [43]. TT homozygotes and CT heterozygotes possess ~1% and ~40% of the enzymatic activity of CC homozygotes, respectively [48,49]. AMP deaminase deficiency affects ~2% of the general population [40,50] and can cause weakness, pain, or cramping in skeletal muscle post-exercise [51,52,53]. Consequently, T-allele carriers show earlier fatigue than CC homozygotes during 30 s Wingate tests and repetitive voluntary isometric contractions [54,55].

The T allele has been reported as under-represented in European elite endurance athletes compared to non-athletes (NAs), although it is unclear whether performance is impaired among T-allele carriers who attain elite status [56]. In 204 Lithuanian endurance and sprint/power athletes, ~75% of the athlete cohort were homozygous for the C allele, with the TT genotype completely absent, compared to ~72% CC homozygotes and ~2% TT homozygotes in NA [57]. To our knowledge, no study has used personal best (PB) run times as a marker of performance; previous studies have instead used variables such as VO_2max_. Similarly, no study has attempted to identify the *AMPD1* C34T polymorphism as a potential marker for elite RU athlete status, or enhanced RU performance. 

Therefore, the first objective of this study was to compare C34T frequency distributions between RU athletes, distance runners (DRs), and NAs. The second objective was to determine whether the C34T genotype was associated with performance data of DRs and RU athletes. It was hypothesised that the TT genotype and T allele would be under-represented in the athlete populations, and that athletes possessing the T allele would exhibit poorer performance than their CC homozygote counterparts.

## 2. Materials and Methods

### 2.1. Participants

The ethics committees of Manchester Metropolitan University, University of Glasgow, University of Cape Town, and University of Northampton granted ethical approval and the study complied with the Declaration of Helsinki [58]. As part of the RugbyGene and GENESIS projects [59,60,61,62], elite male RU athletes, elite and sub-elite male and female DRs, and NAs, all of European ancestry, were recruited after providing written informed consent. The 621 RU athletes (mean (standard deviation) height 1.86 (0.07) m, mass 103 (13) kg) consisted of 59% UK, 11% South African, 12% Irish, 10% Italian, and 8% other nationalities. The 666 DRs (male: height 1.78 (0.06) m, mass 67 (7) kg; female: height 1.65 (0.07) m, mass 54 (5) kg) included 94% UK participants and 6% other nationalities. The 1029 NAs (male: height 1.78 (0.07) m, mass 78 (11) kg; female: height 1.63 (0.07) m, mass 67 (12) kg) comprised 98% UK participants and 2% other nationalities. The DRs were a combination of middle- and long-distance runners (1500 m, 5000 m, 10,000 m, half marathon, and marathon).

### 2.2. Sample Collection

Blood (~65% of all samples), saliva (~20%), or buccal swab samples (~15%) were obtained using previously described protocols [62]. Blood was drawn from a superficial forearm vein into an EDTA tube and stored in sterile tubes at −20 °C until processing. Saliva samples were collected into Oragene DNA OG-500 collection tubes (DNA Genotek, Ottawa, ON, Canada) according to the manufacturer’s protocol and stored at room temperature until processing. Sterile buccal swabs (Omni Swab; Whatman, Springfield Mill, UK) were rubbed against the buccal mucosa of the cheek for ~30 s. Tips were ejected into sterile tubes and stored at −20 °C until processing.

### 2.3. DNA Isolation

DNA isolation was performed at Manchester Metropolitan University, University of Glasgow, University of Cape Town, and University of Northampton laboratories, with some differences between protocols.

At Manchester and Glasgow, DNA isolation was performed with the QIAamp DNA Blood Mini kit and standard spin column protocol, according to the manufacturer’s instructions (Qiagen, West Sussex, UK). In short, 200 μL of whole blood/saliva, or one buccal swab was lysed and incubated, the DNA was washed, and the eluate containing isolated DNA was stored at 4 °C. In Cape Town, DNA was isolated from whole blood by a different protocol [63]. Samples were lysed and centrifuged, the DNA was washed, and the samples were stored at −20 °C. At Northampton, DNA was isolated from whole blood with Flexigene kits (Qiagen). In brief, samples were lysed, and DNA was precipitated and washed, with samples stored at −20 °C.

### 2.4. Genotyping

Samples were genotyped at Manchester Metropolitan University using a protocol described in detail by Heffernan et al. [62] with some variation. For blood and saliva samples, 5 μL GTXpress (Applied Biosystems, Foster City, CA, USA), 4.3 μL nuclease-free H_2_O, 0.5 μL assay mix (rs17602729, GCAGCAAAAGTAATGCAATACTCAC[A/G]TTTCTCTTCAGCTTATGAAGTAAA), and 0.2 μL of purified DNA (~9 ng) were added per well. For buccal swab samples, 5 μL GTXpress, 4 μL nuclease-free H_2_O, 0.5 μL assay mix, and 0.5 μL DNA solution (~9 ng DNA) were added per well. PCR was performed using the StepOnePlus real-time PCR system (Applied Biosystems, Foster City, CA, USA). In brief, denaturation began at 95 °C for 3 s, with 50 cycles of denaturation at 95 °C for 3 s and then annealing and extension at 60 °C for 20 s. Genotyping analysis was performed with StepOnePlus software version 2.3. All samples were analysed in duplicate, with 100% concordance. 

### 2.5. Elite Classification and Proximity Score

RU athletes were considered elite if they had participated in >5 matches since 1995 in the highest professional league in the UK, Ireland, or South Africa [59,61,62]. The ability of DRs was assessed using PB times. Many DRs had recorded PB times across multiple event distances. To permit comparison across events and between male and female athletes, each PB was standardised against a sex- and event-specific reference time. The reference time was defined as the median of the 10 fastest UK performances recorded for the corresponding event and sex, obtained from publicly available records (www.powerof10.info (accessed on 5 May 2025) and https://worldathletics.org (accessed on 5 May 2025)). A Proximity Score (PS) was calculated for each PB as:

PS (%) = ((athlete PB − reference time)/reference time) × 100

The PS represents the percentage difference between an athlete’s PB and the relevant reference time, with lower values indicating superior performance. Positive values indicate slower times than the reference time, negative values indicate faster times, and 0% is indicative of an equivalent time. For athletes with PBs across multiple distances, the lowest PS was used. This enabled comparison across sexes and event distances.

All DRs included had a PB within 33.33% of the median of the 10 best UK times (1948–2025) for their best event. Elite DRs had a PS in the top 50% of the DRs cohort, corresponding to performances within ~17% of the median of the 10 best UK times. These classifications were influenced by the framework of McKay et al. [64], who proposed that an athlete is ‘highly trained/national-level’ within ~20% of world-record performance, which was adapted to the present study (the median times used were ~2–10% slower than world-record times). 

### 2.6. Positional Groups and OPTA Data

To assess the genotype and allele frequencies and compare the performances of the RU athletes, each athlete was allocated to a positional group: forwards (n = 350; props, hookers, locks, flankers, and number eights) and backs (n = 271; scrum halves, fly halves, centres, wingers, and full backs). To account for differences in movement patterns between positions [28], forwards and backs were divided further into positional subgroups: 240 front five, 110 back row, 100 half backs, 75 centres, and 96 back three [62]. 

To assess RU performance, in-game performance metrics, relating to both attacking and defensive play, were obtained using Stats Perform OPTA data (www.statsperform.com, London, UK). Data were collected in real time during fixtures in the highest professional leagues involving clubs from England, Wales, Scotland, Ireland, Italy, and South Africa from the 2012/13 to the 2019/20 seasons. Eleven performance metrics across all eight seasons were obtained for analysis, based on relevance to all positional subgroups. For example, ‘lineouts won’ is not applicable to most backs, so it was not used. Attack metrics consisted of tries, try assists, number of carries, metres gained, metres gained per carry, clean breaks, and defenders beaten. Defence metrics were tackles made, missed tackles, tackle completion percentage, and turnovers won. Appearances, total minutes played, and minutes per appearance represented the athletes’ playing times and were used to normalise the performance metrics per 80 min of playing time.

### 2.7. Statistical Analysis

Statistical analysis was performed using SPSS for Windows version 30 (SPSS, Chicago, IL, USA) software. Due to the low frequency of the TT genotype in subgroup analyses, CC versus T allele carrier comparisons were prioritised where genotype-specific cell counts were small. Genotype, allele-carrier, and allele frequencies were compared using χ^2^ tests. χ^2^ tests of independence were used for between-group comparisons, while goodness-of-fit tests were used where observed distributions were compared with expected distributions. The Kolmogorov–Smirnov test was used to assess normality in OPTA data and PB-derived PS. As the RU data were non-parametric, Kruskal–Wallis H and Mann–Whitney U tests were used to compare in-game performance metrics between genotypes. Conversely, PB-derived PS values were normally distributed; therefore, one-way ANOVA and independent-samples *t*-tests were used to compare PS between genotypes. 

RU performance metrics were weighted to account for differences in positional distribution between genotype groups; one positional group/subgroup was used as the reference group, with the remaining groups weighted to match the positional distribution of the reference group. To control the false discovery rate (5%), Benjamini–Hochberg corrections were applied within 18 predefined families, separated by cohort, subgroup, and metrics tested, with corrected *p* values reported. Genotype distributions were assessed for Hardy–Weinberg equilibrium within the NA, RU, and DRs cohorts. Alpha was set at 0.05.

## 3. Results

Descriptive data for PB times of DRs, medians of the 10 best UK reference times, and athlete counts by event are presented in Table A1, while RU in-game performance metrics by playing position and subgroup are presented in Table A2.

Genotype distributions did not deviate from Hardy–Weinberg equilibrium in the NA, RU, or DRs cohorts (*p* > 0.170). There were no differences in genotype or allele frequencies between the athlete and NA cohorts, nor between RU and DRs (Table 1). There were also no differences in genotype or allele frequencies between NA and RU athletes in each positional group, nor between NA and DRs of each sex or competitive standard (Table 2 and Table 3). There were no TT genotypes among the 100 RU half backs, but at least one TT genotype was present in every other athlete group. Additionally, OPTA data were available for only 44 of the 75 centres, and these data did not include the only centre with the TT genotype. 

**Figure 1:**
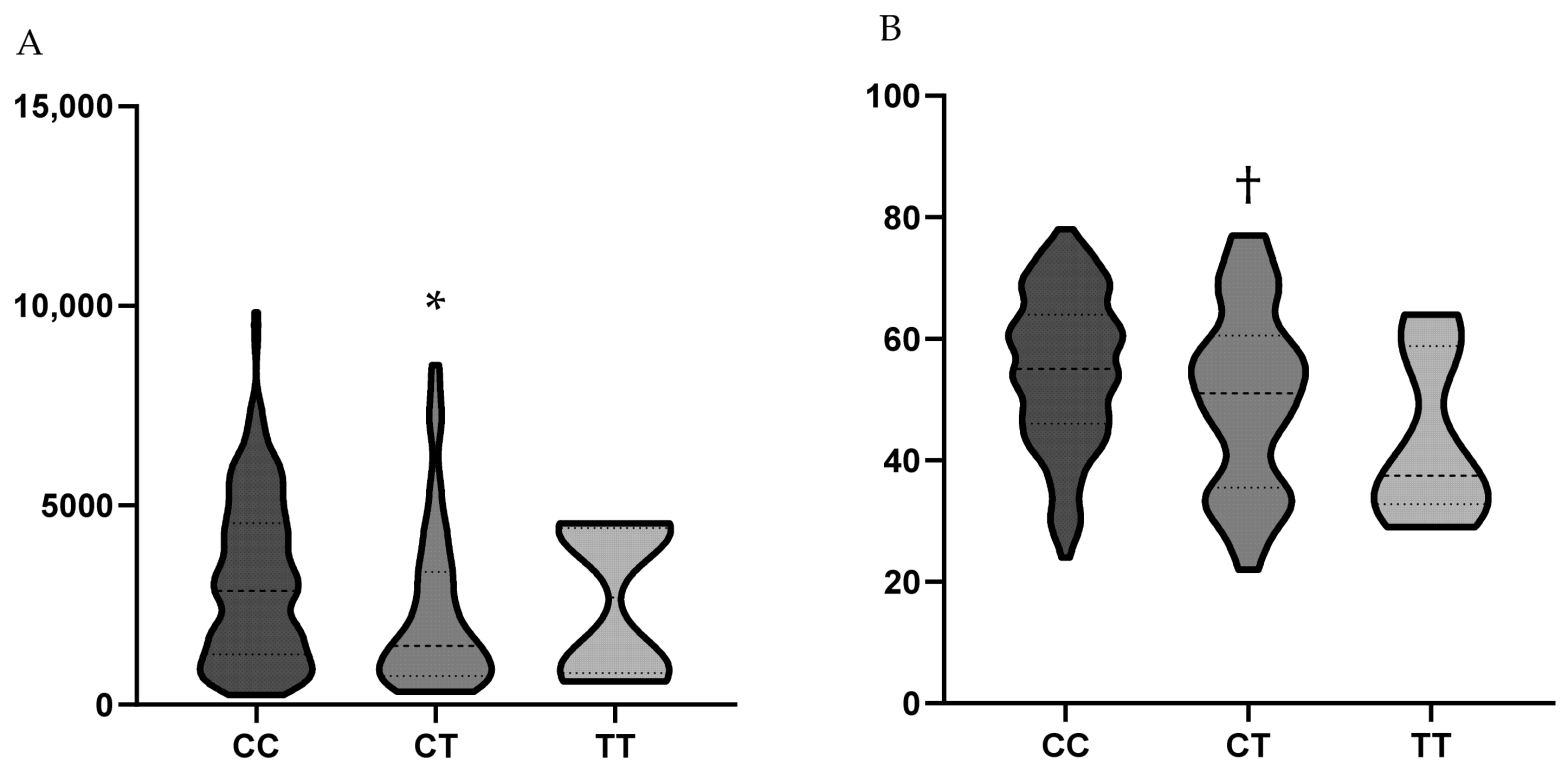
(**A**) Minutes played by RU athletes according to genotype; (**B**) minutes played per appearance by RU athletes according to genotype. Dashed lines represent the 25th, 50th, and 75th percentiles. * Fewer than CC (*p* = 0.003). † Fewer than CC (*p* < 0.001).

Minutes played (*p* = 0.011) and minutes per appearance (*p* = 0.022) were significantly different between genotypes in the RU group, whereas the number of appearances did not differ. RU athletes with the CC genotype played 618 more minutes (mean (SD) 2991 (1969) min vs. 2373 (2057) min, 95% CI of difference = 219–1150, *p* = 0.003, *r* = 0.15; Figure 1A) and six more minutes per appearance (53 (12) min vs. 47 (15) min, 95% CI = 3.3–11.1, *p* < 0.001, *r* = 0.18; Figure 1B) than athletes with the CT genotype, although no differences were observed between CC and TT, nor between CT and TT. Furthermore, athletes with the CC genotype made seven more appearances (55 (33) vs. 48 (30), 95% CI = 2.0–17.0, *p* = 0.028, *r* = 0.13), played 597 more minutes (2991 (1969) min vs. 2394 (2036) min, 95% CI = 302–1303, *p* = 0.006, *r* = 0.17), and six more minutes per appearance (53 (12) min vs. 47 (15) min, 95% CI = 2.4–10.1, *p* = 0.017, *r* = 0.17) than T-allele carriers. 

**Figure 2:**
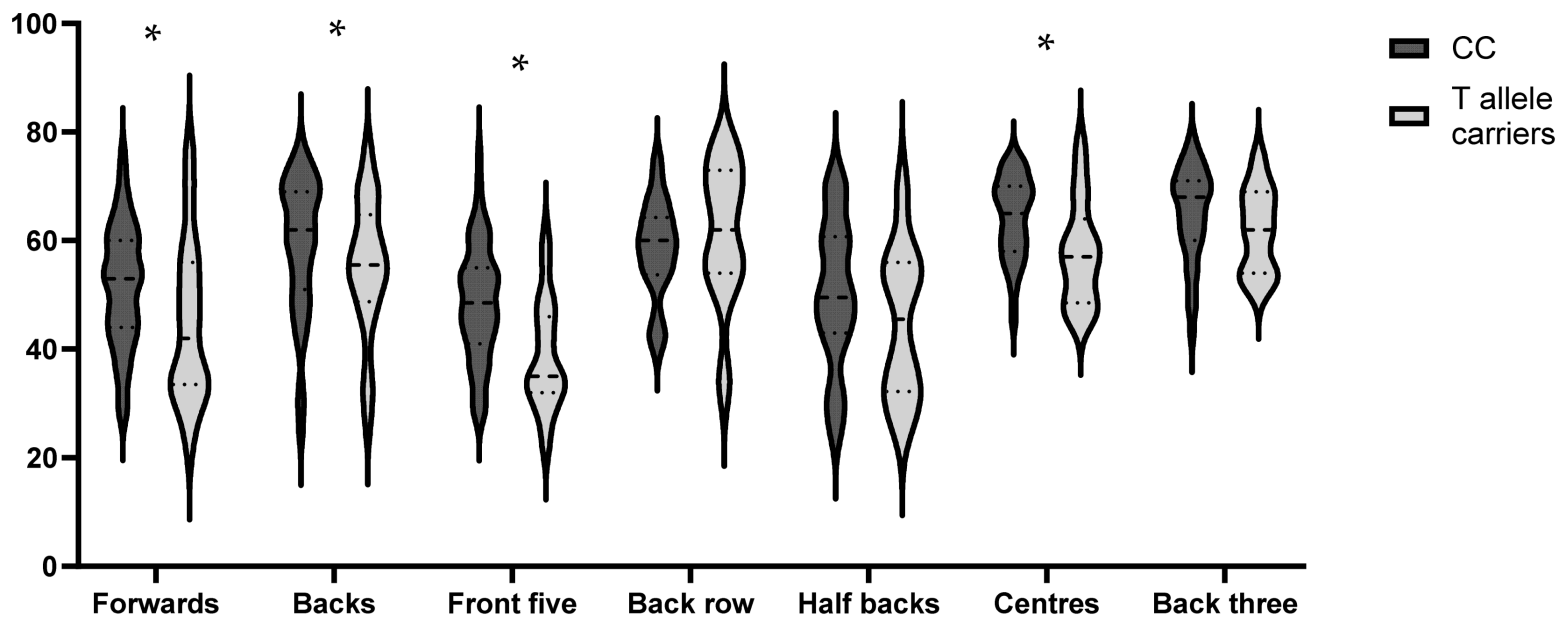
Minutes played per appearance by RU athletes in different playing positions according to possession of the T allele. Dashed lines represent the 25th, 50th, and 75th percentiles. * Greater in CC homozygotes than in T-allele carriers (*p* < 0.05).

Forwards with the CC genotype played 6 more minutes per appearance than T-allele carriers (51 (11) min vs. 45 (15) min, 95% CI of difference = 3.2–13.1, *p* = 0.017, *r* = 0.22; Figure 2). Backs with the CC genotype made 17 more appearances (53 (31) vs. 36 (24), 95% CI = 5.0–27.0, *p* = 0.033, *r* = 0.22), played 1181 more minutes (3242 (2017) min vs. 2061 (1647) min, 95% CI = 399–1937, *p* = 0.017, *r* = 0.24), and played 5 more minutes per appearance (60 (12) min vs. 55 (12) min, 95% CI = 1.1–10.1, *p* = 0.050, *r* = 0.18; Figure 2) than T-allele carriers. Front fives with the CC genotype played 1060 more minutes (2959 (1931) min vs. 1899 (1510) min, 95% CI = 217–1721, *p* = 0.033, *r* = 0.23) and 10 more minutes per appearance (49 (11) min vs. 39 (10) min, 95% CI = 5.7–15.1, *p* = 0.017, *r* = 0.34; Figure 2) than T-allele carriers. However, T-allele carriers achieved 0.09 more clean breaks per 80 min than front five with the CC genotype (0.21 (0.18) vs. 0.12 (0.12), 95% CI = 0.02–0.11, *p* = 0.013, *r* = 0.24). Centres with the CC genotype made 20 more appearances (54 (30) vs. 34 (28), 95% CI = 4.0–39.0, *p* = 0.050, *r* = 0.28) and played 1418 more minutes (3456 (1883) min vs. 2038 (1918) min, 95% CI = 386–2580, *p* = 0.033, *r* = 0.29) than T-allele carriers. There were no significant differences in any of the attacking and defensive metrics between genotypes in the RU, forwards, backs, back row, half backs, centres, or back three groups. 

There were no significant differences in PS between genotypes within the complete DRs cohort. However, PS differed between genotypes in elite DRs (*p* = 0.007). CT athletes were 1.8 percentage points closer to the median of the 10 fastest UK times than CC athletes (9.2 (5.0)% vs. 11.0 (4.2)%; 95% CI of the difference = 0.6–3.0; t(323) = 2.98; Cohen’s d = 0.41; *p* = 0.003) and 5.1 percentage points closer than TT athletes (14.3 (2.8)%; 95% CI = 1.2–8.9; t(70) = 2.63; Cohen’s d = 1.05; *p* = 0.010). CC athletes were also 3.3 percentage points closer to the reference times than TT athletes (95% CI = 0.1–6.4; t(265) = 2.01; Cohen’s d = 0.77; *p* = 0.045).

**Figure 3:**
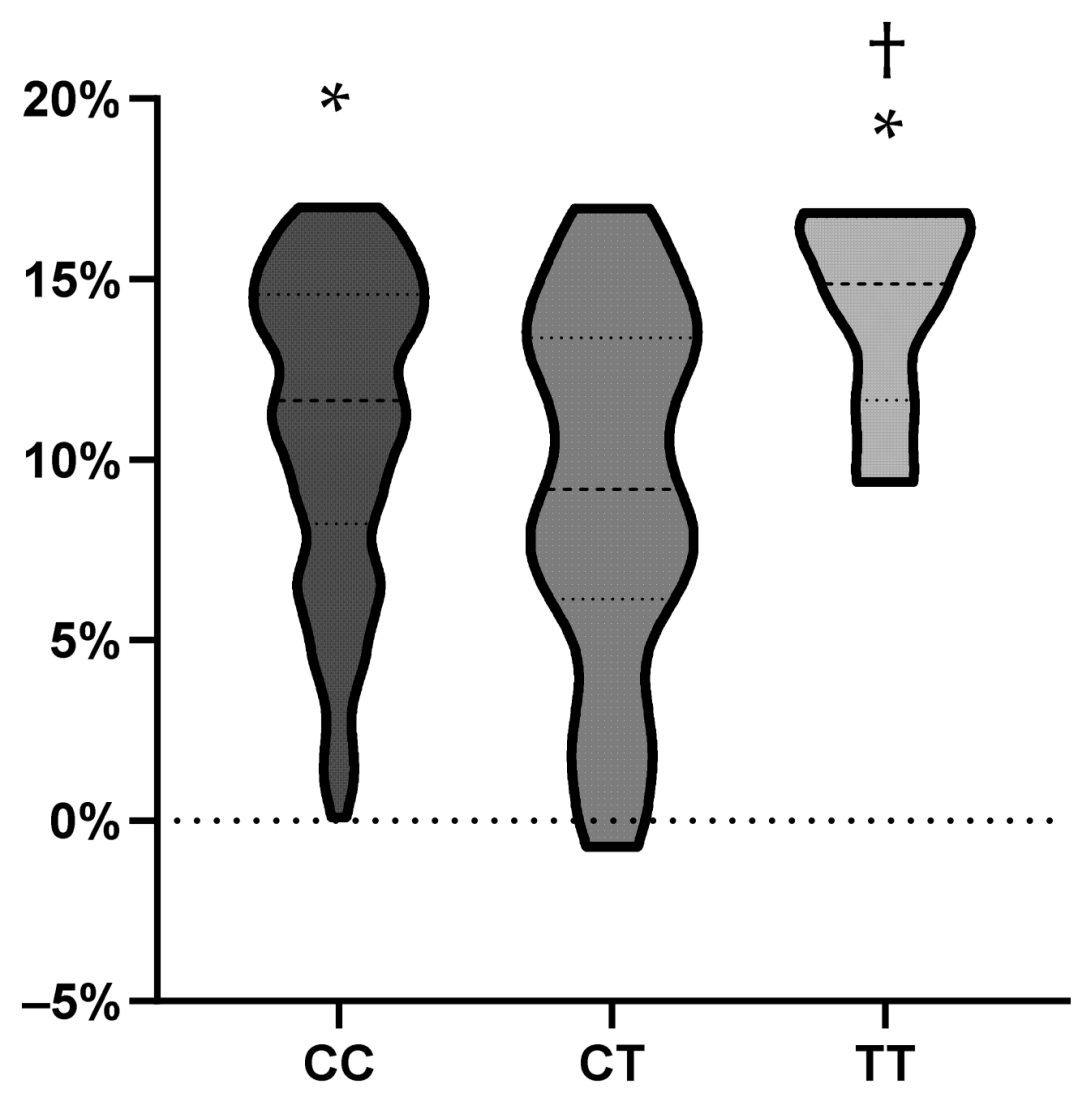
Proximity Score of elite DRs according to genotype. Dashed lines represent the 25th, 50th, and 75th percentiles. * Slower than CT (*p* < 0.05). † Slower than CC (*p* < 0.05).

**Figure 4:**
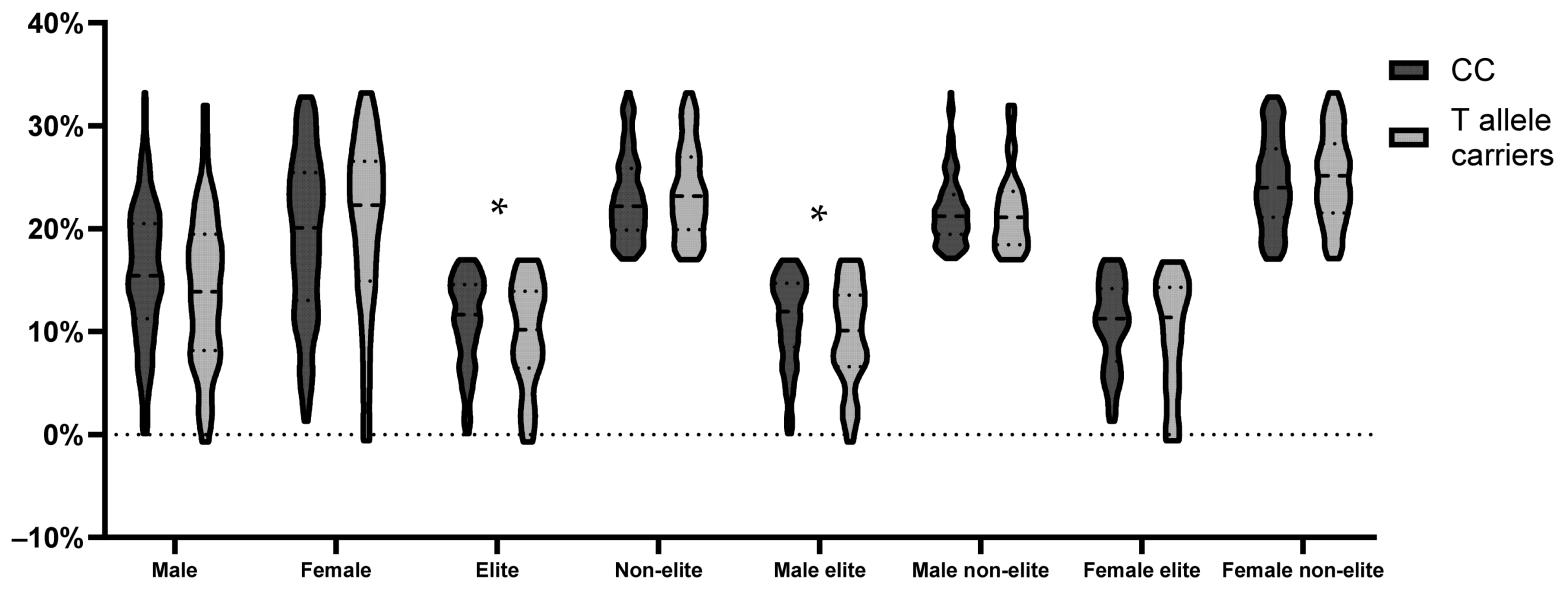
Proximity Score of DRs classified by sex and performance level according to possession of the T allele. Dashed lines represent the 25th, 50th, and 75th percentiles. * CC slower than T-allele carriers (*p* < 0.05).

PS also differed between genotypes in elite male DRs (*p* = 0.014). CT athletes were 2.1 percentage points closer to the reference times than CC athletes (9.1 (4.7)% vs. 11.2 (4.3)%; 95% CI = 0.6–3.4; t(229) = 2.84; Cohen’s d = 0.46; *p* = 0.005) and 5.2 percentage points closer than TT athletes (14.3 (3.1)%; 95% CI = 1.2–9.2; t(51) = 2.60; Cohen’s d = 1.13; *p* = 0.012). However, no difference was observed between CC and TT athletes. PS values for the DRs subgroups are shown in Figure 3 and Figure 4.

## 4. Discussion

Although the C34T *AMPD1* polymorphism has previously been reported as under-represented in athletic cohorts [56], we found no association with athlete status in RU or DRs, or with playing position in RU. To our knowledge, this is the first study to examine associations between *AMPD1* C34T genotype and RU in-game performance or PB-derived DRs performance. Several exploratory associations were observed between genotype and RU playing time and DRs performance, while attacking and defensive metrics were largely unaffected apart from there being more clean breaks among front five T-allele carriers.

The T allele has previously been reported as under-represented in endurance and strength/power athletes compared with non-athletes [56,65], whereas no such differences were observed in the present study. Genotype and allele frequencies also did not differ between RU and DRs athletes, contrasting with reports of a higher CC genotype frequency in sprint/power than endurance athletes [57]. However, RU combines substantial aerobic and anaerobic demands and cannot be classified solely as a sprint/power sport [32]. All cohorts were of European ancestry, in which the T-allele frequency is approximately 12%, compared with 0.1–0.5% in African and East Asian populations [66].

AMP deaminase deficiency may alter skeletal muscle energy metabolism without uniformly impairing exercise capacity. Reduced ATP degradation [67] and increased post-exercise adenosine accumulation [49] have been reported, while greater reliance on oxidative metabolism has been proposed [49]. Phosphocreatine hydrolysis, lactate accumulation, and TCA cycle anaplerosis may remain unaffected [67,68]. These compensatory responses may partly explain the inconsistent genotype–performance associations observed across RU and DRs.

RU athletes with the CC genotype accumulated between 25% and 70% greater total playing time and up to 26% more minutes per appearance than T-allele carriers. This may be consistent with differences in fatigue resistance, although playing time is not a direct measure of physiological capacity. Fewer minutes per appearance among T-allele carriers could reflect earlier or more frequent substitutions due to fatigue suggested by real-time performance metrics or otherwise perceived by coaching staff; however, selection and substitution are also influenced by tactics, injury history, squad role, selection policy, and international availability. Although starter status has been associated with superior physical characteristics in under-16 rugby players [69], experience, technical ability, and tactical suitability are also likely to influence selection. The T allele has previously been associated with injury risk in endurance athletes [70], suggesting that injury-related availability could represent an additional explanation for the observed differences. However, injury incidence and reasons for substitution were not assessed in the present study, and the mechanisms underlying these associations therefore remain uncertain.

An additional association was observed within front five, where T-allele carriers recorded more clean breaks than those with the CC genotype. This finding is difficult to reconcile with the playing-time results already discussed and should be interpreted cautiously. Clean breaks are highly context-dependent and were markedly less frequent among the front five group than in other positional subgroups, consistent with their game-specific roles. Consequently, small absolute differences may have produced comparatively large relative differences within this subgroup. Given that the association was isolated to a single positional subgroup and performance outcome, it may reflect positional, tactical, or sampling variation rather than a consistent genotype-related effect. Although the possibility of a genuine association cannot be excluded, replication in larger position-specific cohorts is required. 

Elite and elite male DRs with the CT genotype had PSs ~2 percentage points lower, and therefore superior, than DRs with the CC genotype, whereas differences between CC and TT genotypes were uncommon. These findings do not support the interpretation that possession of the T allele uniformly impairs endurance performance. A case report by Lucia et al. [71] described an elite CT runner who had lower height, body mass, and body mass index, an approximately 11% higher VO_2max_, and lower lactate and ammonia concentrations than comparison cohorts of Spanish and African CC runners. As AMP deamination produces IMP and ammonia [40,43,44], reduced AMP deaminase activity may lower ammonia production during exercise [48,49,55]. Lower circulating ammonia could theoretically reduce cerebral ammonia uptake and its effects on neurotransmission, energy metabolism, and central fatigue [72,73], although these mechanisms were not assessed in the present study. CT heterozygotes retain approximately 40% of the enzyme activity of CC homozygotes, whereas TT homozygotes are almost entirely deficient [48,49]. Partial enzyme activity, lower ammonia production, favourable anthropometry, and high cardiorespiratory fitness may therefore have contributed to the reported athlete’s performance. However, evidence from a single athlete does not establish a heterozygote advantage. Likewise, the limited differences between CC and TT genotypes across DRs outcomes in the current study suggest that AMP deaminase deficiency is not consistently reflected in endurance performance, although the small number of TT athletes and variation in training status, event specialisation, and other physiological characteristics limit interpretation.

The principal strengths of the present study were the large overall cohorts relative to previous single-sport genetic studies, assembled through the RugbyGene and GENESIS projects [59,60,61,62], and the use of objective, sport-specific performance data. The Proximity Score enabled comparisons across running events and sexes, while the substantial volume of OPTA data permitted assessment of playing time and in-game performance across several seasons. Positional stratification and correction for multiple comparisons further strengthened the analyses. Nevertheless, the findings should be interpreted in the context of several important limitations.

Most genotype comparisons were not statistically significant, and the positive findings were confined to a small number of exploratory subgroup analyses across numerous outcomes. Subdivision by genotype, sex, performance level, and playing position also substantially reduced sample sizes, particularly for the uncommon TT genotype, limiting the precision and statistical power of some genotype-specific comparisons. Although the false discovery rate was controlled, the possibility that some isolated associations represent chance findings cannot be excluded. In addition, OPTA data were available for only 347 of the 621 RU athletes and covered the 2012/13 to 2019/20 seasons, meaning that athletes contributed differing amounts of observable career time. Playing time is also influenced by injuries, coaching decisions, substitutions, tactics, squad role, selection policy, and international availability and should therefore not be interpreted as a direct measure of fatigue resistance or physiological capacity. The observed effect sizes were generally modest; where larger effects were observed in comparisons involving the TT genotype, the estimates were based on small groups and were therefore imprecise. Their practical relevance to elite athletic performance remains uncertain. Furthermore, the proposed metabolic explanations were not directly examined and should be regarded as hypotheses rather than established mechanisms. Accordingly, the positive associations should be considered preliminary and replication in larger, independent cohorts would be valuable, although the rarity of the TT genotype makes such replication challenging.

In summary, genotype and allele frequencies did not differ between athletes and NA or between sport groups, providing no evidence that the *AMPD1* C34T polymorphism is associated with athlete status in either RU or DRs athletes. Furthermore, most performance outcomes across both cohorts were unrelated to genotype. However, the CC genotype was associated with greater playing time in several RU groups and subgroups, whereas isolated favourable outcomes were observed among T-allele carriers in specific RU and DRs comparisons. These associations arose within predominantly negative statistical results, and their practical significance remains uncertain. Although greater RU playing time per competitive appearance is compatible with greater fatigue resistance, playing time is not a direct measure of physiological capacity and the underlying mechanisms cannot be determined from the present data.

## Figures and Tables

**Table 1:** Genotype and allele distribution of athletes according to sport and NAs, presented as genotype/allele count followed by percentage in parentheses. No significant differences were observed between frequencies.

Genotype	Athletes N = 1287	DRs N = 666	RU N = 621	NA N = 1029
CC	993 (77.2)	519 (77.9)	474 (76.3)	792 (77.0)
CT	276 (21.4)	138 (20.7)	138 (22.2)	216 (21.0)
TT	18 (1.4)	9 (1.4)	9 (1.4)	21 (2.0)
C-allele carriers	1269 (98.6)	657 (98.6)	612 (98.6)	1008 (98.0)
T-allele carriers	294 (22.8)	147 (22.1)	147 (23.7)	237 (23.0)
C allele	2262 (87.9)	1176 (88.3)	1086 (87.4)	1800 (87.5)
T allele	312 (12.1)	156 (11.7)	156 (12.6)	258 (12.5)

DRs = distance runners, RU = rugby union, NA = non-athlete.

**Table 2:** Genotype and allele distribution of RU athletes according to positional group and subgroup, presented as genotype/allele count followed by percentage in parentheses. No significant differences were observed between frequencies.

Genotype	Forwards N = 350	Backs N = 271	Front Five N = 240	Back Row N = 110	Half Backs N = 100	Centres N = 75	Back Three N = 96
CC	273 (78.0)	201 (74.2)	187 (77.9)	86 (78.2)	79 (79.0)	50 (66.7)	72 (75.0)
CT	70 (20.0)	68 (25.1)	48 (20.0)	22 (20.0)	21 (21.0)	24 (32.0)	23 (24.0)
TT	7 (2.0)	2 (0.7)	5 (2.1)	2 (1.8)	0 (0.0)	1 (1.3)	1 (1.0)
C-allele carriers	343 (98.0)	269 (99.3)	235 (97.9)	108 (98.2)	100 (100.0)	74 (98.7)	95 (99.0)
T-allele carriers	77 (22.0)	70 (25.8)	53 (22.1)	24 (21.8)	21 (21.0)	25 (33.3)	24 (25.0)
C allele	616 (88.0)	470 (86.7)	422 (87.9)	194 (88.2)	179 (89.5)	124 (82.7)	167 (87.0)
T allele	84 (12.0)	72 (13.3)	58 (12.1)	26 (11.8)	21 (10.5)	26 (17.3)	25 (13.0)

**Table 3:** Genotype and allele distribution of DRs according to sex and performance level, presented as genotype/allele count followed by percentage in parentheses. No significant differences were observed between frequencies.

Genotype	Elite N = 332	Sub-Elite N = 334	Male Elite N = 237	Male Sub-Elite N = 167	Female Elite N = 95	Female Sub-Elite N = 167
CC	260 (78.3)	259 (77.5)	184 (77.6)	137 (82.0)	76 (80.0)	122 (73.1)
CT	65 (19.6)	73 (21.9)	47 (19.8)	29 (17.4)	18 (18.9)	44 (26.3)
TT	7 (2.1)	2 (0.6)	6 (2.5)	1 (0.6)	1 (1.1)	1 (0.6)
C-allele carriers	325 (97.9)	332 (99.4)	231 (97.5)	166 (99.4)	94 (98.9)	166 (99.4)
T-allele carriers	72 (21.7)	75 (22.5)	53 (22.4)	30 (18.0)	19 (20.0)	45 (26.9)
C allele	585 (88.1)	591 (88.5)	415 (87.6)	303 (90.7)	170 (89.5)	288 (86.2)
T allele	79 (11.9)	77 (11.5)	59 (12.4)	31 (9.3)	20 (10.5)	46 (13.8)

## Data Availability

Data are available for research purposes upon reasonable request to the corresponding author.

## Abbreviations

The following abbreviations are used in this manuscript:

ADP	Adenosine diphosphate
AMP	Adenosine monophosphate
*AMPD1*	Adenosine monophosphate deaminase 1
ATP	Adenosine triphosphate
BiP	Ball-in-play
DRs	Distance runners
NA	Non-athletes
PB	Personal best
PS	Proximity score
RU	Rugby union
VO_2max_	Maximal rate of oxygen uptake

## Appendix A

**Table A1:** Median of the 10 best UK times ever achieved, athlete count and personal best times of DRs according to event (M = male, F = female).

Metric	1500 m		5000 m		10,000 m		Half Marathon		Marathon	
	M	F	M	F	M	F	M	F	M	F
Median of the 10 best UK times	00:03:30	00:03:58	00:13:02	00:14:46	00:27:25	00:30:53	01:00:35	01:07:10	02:08:09	02:23:17
N	61	45	30	18	15	11	154	85	144	103
Range	00:01:08	00:01:06	00:02:53	00:04:08	00:08:44	00:07:14	00:18:39	00:17:32	00:37:41	00:47:22
Mean	00:03:59	00:04:38	00:14:38	00:16:46	00:29:12	00:34:06	01:11:06	01:22:32	02:28:01	02:51:47
SD	00:00:14	00:00:16	00:00:44	00:01:13	00:02:06	00:02:06	00:03:39	00:04:08	00:08:19	00:12:00
Median	00:03:58	00:04:37	00:14:35	00:16:35	00:28:48	00:33:58	01:11:46	01:22:48	02:28:06	02:54:10

**Table A2:** In-game performance metrics of RU athletes according to playing position and subgroup, presented as mean (SD).

Metric	All	Forwards	Backs	Front Five	Back Row	Half Backs	Centres	Back Three
N	347	197	150	137	60	54	44	52
Appearances	52 (32)	55 (33)	49 (30)	56 (33)	53 (33)	54 (33)	48 (31)	44 (27)
Minutes played	2923 (2031)	2907 (2046)	2944 (2017)	2750 (1898)	3266 (2327)	2904 (2144)	3037 (1982)	2906 (1945)
Minutes per appearance	53.8 (13.0)	50.4 (12.2)	58.3 (12.6)	46.7 (11.3)	58.9 (9.7)	49.2 (13.6)	62.4 (8.7)	64.4 (8.4)
Tries per 80 min	0.13 (0.14)	0.09 (0.11)	0.19 (0.16)	0.08 (0.11)	0.12 (0.10)	0.16 (0.17)	0.14 (0.09)	0.25 (0.17)
Try assists per 80 min	0.10 (0.13)	0.03 (0.04)	0.18 (0.15)	0.03 (0.04)	0.04 (0.05)	0.29 (0.15)	0.12 (0.09)	0.13 (0.13)
Carries per 80 min	7.3 (2.5)	7.4 (3.0)	7.2 (1.8)	6.5 (2.6)	9.4 (2.9)	6.5 (1.8)	7.1 (1.7)	8.1 (1.7)
Metres gained per 80 min	21.9 (15.9)	13.2 (9.9)	33.3 (15.0)	9.2 (5.7)	22.4 (11.4)	25.8 (9.3)	25.9 (7.9)	47.5 (14.1)
Metres gained per carry	2.9 (1.8)	1.7 (0.8)	4.5 (1.4)	1.4 (0.6)	2.3 (0.7)	4.0 (0.9)	3.7 (1.0)	5.8 (1.1)
Clean breaks per 80 min	0.40 (0.35)	0.19 (0.17)	0.67 (0.33)	0.14 (0.14)	0.31 (0.18)	0.56 (0.29)	0.56 (0.23)	0.90 (0.34)
Defenders beaten per 80 min	0.98 (0.82)	0.60 (0.63)	1.48 (0.78)	0.40 (0.39)	1.06 (0.82)	1.23 (0.72)	1.23 (0.44)	1.97 (0.83)
Tackles made per 80 min	8.4 (3.2)	10.4 (2.5)	5.8 (2.0)	9.9 (2.4)	11.5 (2.4)	6.3 (1.5)	7.3 (1.7)	4.1 (1.5)
Missed tackles per 80 min	1.2 (0.4)	1.1 (0.4)	1.2 (0.4)	1.1 (0.4)	1.2 (0.4)	1.4 (0.4)	1.2 (0.4)	1.1 (0.4)
Tackle completion (%)	86.4 (6.3)	90.0 (3.9)	81.7 (5.8)	89.7 (4.0)	90.7 (3.7)	81.4 (5.2)	85.3 (4.2)	79.0 (6.1)
Turnovers won per 80 min	0.40 (0.30)	0.44 (0.35)	0.34 (0.20)	0.33 (0.24)	0.69 (0.43)	0.26 (0.18)	0.45 (0.20)	0.32 (0.18)
